# Case Report: Guillain-Barré Syndrome as Primary Presentation of Systemic Lupus Erythematosus (SLE-GBS) in a Teenage Girl

**DOI:** 10.3389/fped.2022.838927

**Published:** 2022-03-17

**Authors:** Elham Beshir, Ernestina Belt, Nidheesh Chencheri, Aqdas Saqib, Marco Pallavidino, Ulrich Terheggen, Abdalla Abdalla, Leal Herlitz, Elsadeg Sharif, Martin Bitzan

**Affiliations:** ^1^Department of Paediatrics, Al Jalila Specialty Children's Hospital, Dubai, United Arab Emirates; ^2^College of Medicine, Mohammed Bin Rashid University of Medicine and Health Sciences, Dubai, United Arab Emirates; ^3^Department of Anatomic Pathology, Cleveland Clinic, Cleveland, OH, United States

**Keywords:** Guillain-Barré syndrome (GBS), systemic lupus erythematosus (SLE), plasma exchange therapy, rituximab, intravenous immunoglobulin (IVIg) therapy, pediatric lupus nephritis, tracheostomy, B cell depletion therapy

## Abstract

Peripheral nervous system involvement accounts for fewer than 10% of SLE cases with neuropsychiatric manifestations. Guillain-Barré syndrome (GBS) as the presenting, major manifestation of pediatric SLE is extremely rare, and the best treatment approach is unknown. A 14-year-old, previously healthy female teenager developed classic features of GBS with ascending bilateral muscle weakness leading to respiratory insufficiency, associated with protein-cell dissociation in cerebro-spinal fluid, nerve root enhancement by MRI and reduction in compound muscle action potential amplitude. SLE was diagnosed serologically and histologically (lupus nephritis WHO class II). Despite immediate treatment with intravenous immunoglobulin (IVIg), methylprednisolone pulses and subsequently, rituximab, the patient required prolonged mechanical ventilation. She achieved full recovery following 14 PLEX treatments and two more rituximab infusions. Anti-dsDNA, C3, C4 and urinalysis normalized while anti-Smith and Sjögren antibodies persisted 15 months after disease onset, with no other lupus manifestations. Review of the literature revealed two pediatric cases of GBS at the onset of SLE and a third case with GBS 6 years after the diagnosis of SLE. Conventional GBS therapy may not be adequate to treat SLE-GBS. SLE should be included in the differential diagnosis of GBS. Importantly, treatment experiences and outcomes of such cases need be reported to inform future treatment recommendations.

## Introduction

Guillain-Barré syndrome (GBS) has been defined as an acute autoimmune polyradiculoneuritis affecting the peripheral nervous system [acute inflammatory polyradiculoneuropathy (AIPN)]. The classical description refers to the landmark paper by Guillain et al. (1). “GBS” syndrome (2) is thought to be a post-infectious autoimmune disease, resulting from the immune-mediated injury of myelin and/or axonic neurons; the contribution of genetically mediated dysregulation of inflammation and immune responses has been postulated [summarized in (3, 4)]. GBS has been described occasionally in systemic lupus erythematosus (SLE) patients (5–8), generally without preceding infection. Incidence estimates of “classical” (non-SLE related) GBS are 0.5–2 cases per 100,000 population per year (9). The disease peaks during adolescence and in older age and appears to affect males more often than females (3, 10). Fatal outcome has been reported (11–13).

SLE is an autoimmune disease with multi-organ involvement and substantial morbidity. Its etiology involves genetic, epigenetic, and environmental factors (14). The initial diagnosis of SLE can pose diagnostic challenges due to its variable presentation. The incidence of neuropsychiatric symptoms (NPSLE) among children with SLE ranges from 14 to 75% (8, 15). Peripheral nervous system involvement, including GBS, accounts for <10% of NPSLE cases (7, 8). CNS symptoms rarely precede the onset of typical features of SLE (5). Among the few reported cases of SLE with GBS as initial presentation, only two were in the pediatric age group (16, 17). In another pediatric case of SLE-GBS (18), the neurological complication developed 6 years after the lupus diagnosis. The exact pathogenic mechanism remains unknown, and a consensus about the treatment of SLE-GBS has still to emerge.

Here, we describe a third pediatric patient with GBS as the initial manifestation of SLE. She experienced progressive, ascending muscle weakness that led to the diagnosis of GBS, while lupus was diagnosed on the basis of laboratory tests and renal biopsy during the acute GBS presentation. We further detail the interdisciplinary diagnostic and therapeutic approach which resulted in the full recovery of the patient.

This case is unique in that GBS is a rare, but clinically dramatic complication of (pediatric) SLE. Here GBS preceded the recognition of (mild) lupus. A detailed description of clinical presentation, therapy, and outcome is of practical relevance to inform future management recommendations. This case and a comparison of previous pediatric (and adult) reports suggest that SLE-GBS and classical GBS require a modified treatment approach.

## Case Report

A 14-year-old previously healthy teenager presented with a 4-week history of numbness and paresthesia in both legs, 10 days of disparate eye movements with subjective rightward-gaze diplopia, and 6-8 days of ascending, bilateral muscle weakness, more pronounced on the left. She had not been able to walk for 7 days when she presented to our Emergency Department and had developed mild swallowing difficulties, a temperature of 38.1°C, and left-sided knee and hip pain 2 days prior to admission.

She denied fever or vaccination preceding the onset of neuromuscular symptoms. Specifically, there was no diarrhea or vomiting, signs of upper respiratory infection, headache or seizures, rash, edema, joint pain, or macroscopic hematuria. The family also denied changes in her mood, behavior or academic performance. No constipation or urinary retention was reported. She menstruated regularly, last 3 weeks prior to hospitalization.

There was no history of sick contacts, including COVID-19, or recent travel outside the country. She was fully immunized according the national schedule, including polio and quadrivalent *N. meningitidis* vaccines.

### Family History

Family history is significant for a paternal aunt with a presumptive diagnosis of SLE. Several members of the paternal family have hypothyroidism and glucose-6-phosphate dehydrogenase (G6PD) deficiency. The patient's older sister has been treated for hyperthyroidism. There is type 2 diabetes mellitus and arterial hypertension in elderly members of the family. Consanguinity was denied.

### Clinical Examination

At the time of admission, the patient was bed-bound due to severe generalized weakness but was conscious and alert. She complained of diplopia and swallowing difficulty. Cranial nerve examination showed right eye convergent squint and bilateral gaze palsy, right more than left. The pupils were equal and reactive. There was no ptosis or facial muscle weakness, but she had bulbar symptoms and a weak gag reflex. Shoulder and arm movements were weak, corresponding to Medical Research Council (MRC) Muscle Scale 1-2/5 (19). Dorsiflexion at the wrist against gravity was 3/5, and the palmar grasp was weak. The lower limbs also demonstrated significant proximal weakness with MRC scale of 1/5 at hip and knee joint movements. The ankle dorsiflexion and plantar flexion appeared less affected with MRC scale of 3-4/5. Overall, the left side appeared weaker than the right. She was very hypotonic, with total areflexia. She complained of paresthesia, but there was no sensory impairment, cerebellar signs or meningeal irritation. At presentation, she had no vasculitic lesions, arthralgia, cardiac, or other system abnormalities.

### Laboratory Findings

Initial laboratory findings included a mildly decreased absolute lymphocyte count, normal creatinine clearance (eGFR 144 ml/min/1.73 m^2^), and elevated erythrocyte sedimentation rate with only slightly elevated C-reactive protein (CRP). Screening for an autoimmune etiology of her presentation revealed decreased CH50, low C3 and C4 serum levels, elevated antinuclear (ANA) and anti-dsDNA antibodies, and rheumatoid factor (Table 1). Creatine kinase, acetylcholine receptor antibody, thyroid function and B12 levels were normal.

**Table 1:** Inflammatory markers and lupus serology ^a^.

**Test**		**Initial results**	**In remission**	**Reference range**
**Cerebrospinal fluid findings** ^**b**^				
Cell count		3 ×10^6^/L	ND	0−7 ×10^6^/L
Total protein		63 mg/dl	ND	0.0-44.0 mg/dl
IgG		22.9 mg/dl	ND	0.0-8.6 mg/dl
IgA		1.25 mg/dl	ND	0.00-0.64 mg/dl
IgM		0.14 mg/dl	ND	0.00-0.26 mg/dl
Gamma globulin		27.5%	ND	3-13%
M spike		Negative	ND	Negative
Oligoclonal bands		Negative	ND	Negative
Anti-NMDAR/NMO/MOG ^c^		Negative	ND	Negative
**Inflammatory markers in serum**				
CRP		11.6 mg/L	0.7 mg/dL	<5 mg/L
ESR		92 mm/h	10 mm/h	<15 mm/h
Fibrinogen		548 mg/dl	ND	212-433 mg/dl
Ferritin		108 ng/ml	39 ng/mL	12-68 ng/ml
IL-1 beta		<2.9 pg/ml	ND	<3.0 pg/ml
IL-10		20.7 pg/ml	ND	3.7-23.3 pg/ml
TNF-alpha		4.0 pg/ml	ND	0.0-2.2 pg/ml
IL-6		14.2 pg/ml	ND	<7 pg/ml
**Lupus serology**				
ANA		1:1280 (speckled)	ND	Negative
Anti-dsDNA antibody		12 IU/ml	<1 IUml	0-4 IU/ml
dsDNA Crithidia IFA		ND	Negative	Negative
Rheumatoid factor		79 IU/ml	ND	≤ 13 IU/ml
C3		43 mg/dl	142 mg/dl	83-193 mg/dl
C4		8.7 mg/dL	43.4 mg/dl	15-57 mg/dl
CH50		28 U/ml	ND	>41 U/ml
Lupus anticoagulant		Negative	ND	Negative
RNP antibody		>8.0 AI	Positive	0.0-0.9 AI
Smith antibody		>8.0 AI	>8.0 AI	0.0-0.9 AI
Scleroderma-70 antibody		<0.2 AI	Negative	0.0-0.9 AI
Sjogren's SS-A (Ro) antibody		>8.0 AI	>8.0 AI	0.0-0.9 AI
Sjogren's SS-B (La) antibody		>8.0 AI	>8.0 AI	0.0-0.9 AI
Chromatin antibody		>8.0 AI	>8.0 AI	0.0-0.9 AI
Ribosomal P antibody		0.6 AI	1.4 AI	0.0-0.9 AI
Anti-Jo-1		<0.2 AI	Negative	0.0-0.9 AI
Anti-GM1 (IgG)		6%	ND	0-30 %
**Urine studies**				
Hematuria	Day 0	0-3 RBC/HPF	0-3 RBC/HPF	0-3 RBC/HPF
RBC microscopy	Day 13	15-20 RBC/HPF		
Proteinuria	Day 0	1 + (0.3 g/L) ^d^	Negative	Negative
	Day 14	2.20 g/g ^e^	0.12 g/g	<0.20 g/g

*^a^Samples were obtained at presentation or during the first days of illness.*

*^b^CSF concentrations of alpha-1, alpha-2, and beta globulin, albumin and pre-albumin fractions were normal, as were the lactic acid and Cl^−^ concentrations.*

*^c^Anti-MOG (myelin oligodendrocyte glycoprotein), NMDA (https://en.wikipedia.org/wiki/N-methyl_D-aspartate_receptorN-methyl D-aspartate) receptors and NMO (neuromyelitis optica)/AQP4 (aquaporin 4) IgG antibodies were undetectable.*

*^d^Urine dipstick.*

*^e^Quantitative urine protein creatinine ratio.*

*IFA, immunofluorescence assay; RBC, red blood cells*.

Subsequently, antibodies to extractable nuclear antigens (ENA), including ribonucleoprotein (RNP), chromatin, Smith and SS-A (Ro)/SS-B (La), were detected in high concentrations. The initial urine sample showed a small amount of protein. Hematuria and proteinuria peaked about 2 weeks after admission (Table 1).

**Figure 1:**
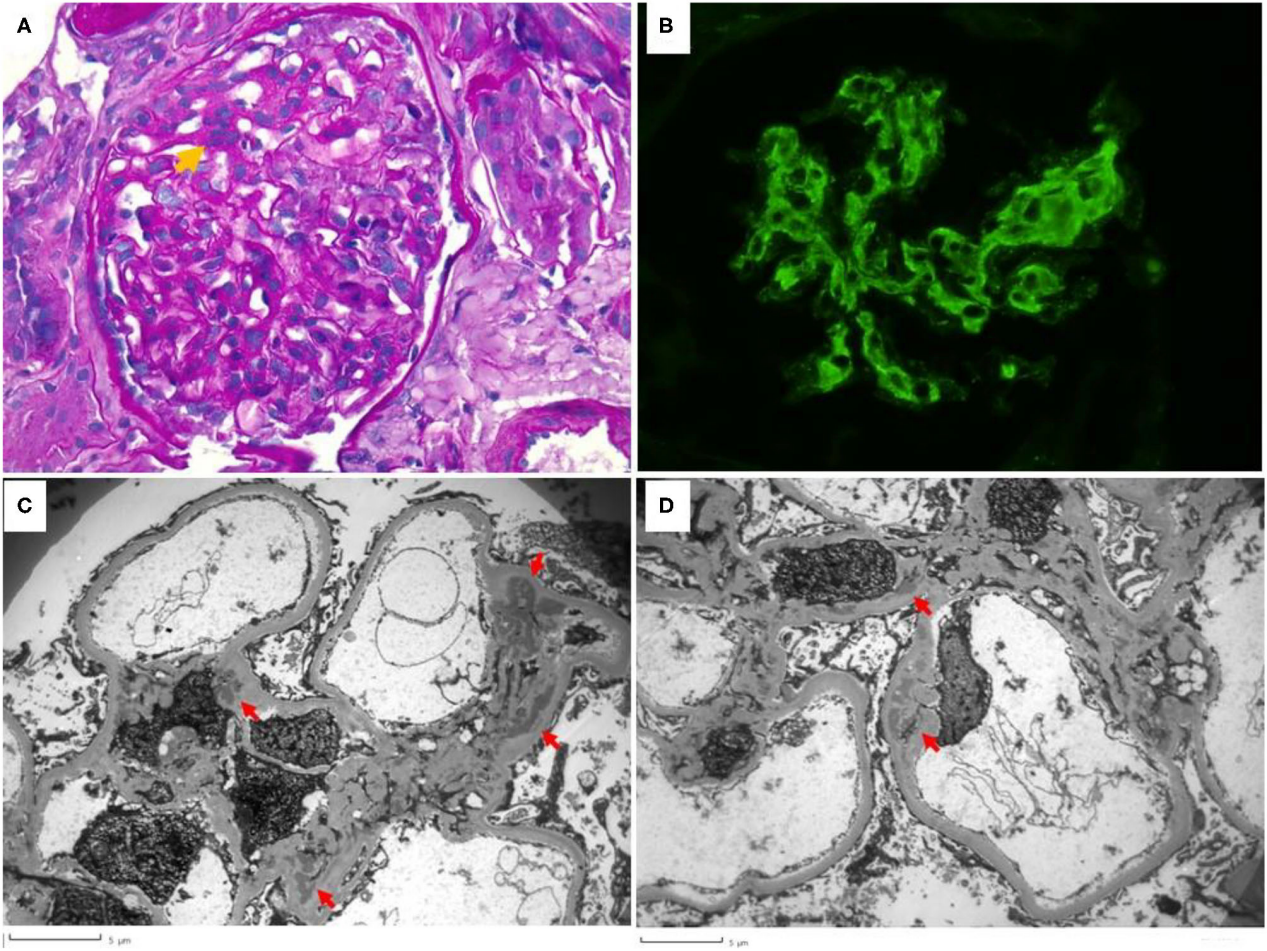
Renal biopsy. **(A)** shows segmental mesangial hypercellularity (orange arrow) with >3 mesangial cells in a mesangial area. Peripheral capillaries are patent and no lesions of endocapillary hypercellularity or crescent formation was seen in the biopsy (Periodic Acid Schiff, 400× magnification). **(B)** shows the granular mesangial immune complex deposition that stained 2+ for IgG, IgA, C3, C1q, kappa and lambda (IgG is shown, 400× magnification). **(C,D)** highlight the electron microscopy findings. Electron dense deposits (red arrows) are present globally in mesangial areas but are not seen to involve the subepithelial or subendothelial distributions. Podocyte foot process effacement is mild.

The laboratory results were suggestive of SLE. A kidney biopsy was performed on day 8 of admission; results were consistent with lupus nephritis WHO class II, with a “full house” immunofluorescence pattern (20) (Figure 1).

Cerebrospinal fluid analysis prior to treatment revealed protein-cell dissociation with an increased total protein concentration, mainly due to IgG and IgA and only 3 × 10^6^/L mononuclear cells (lymphocytes and monocytes) (Table 1). Sequential COVID-19 PCR tests over the first 2 ½ weeks and SARS-CoV-2 specific IgG antibody assays remained negative. CMV IgG was elevated, but CMV IgM, EBV serology, and Mycoplasma IgM results were negative.

### Imaging Studies

**Figure 2:**
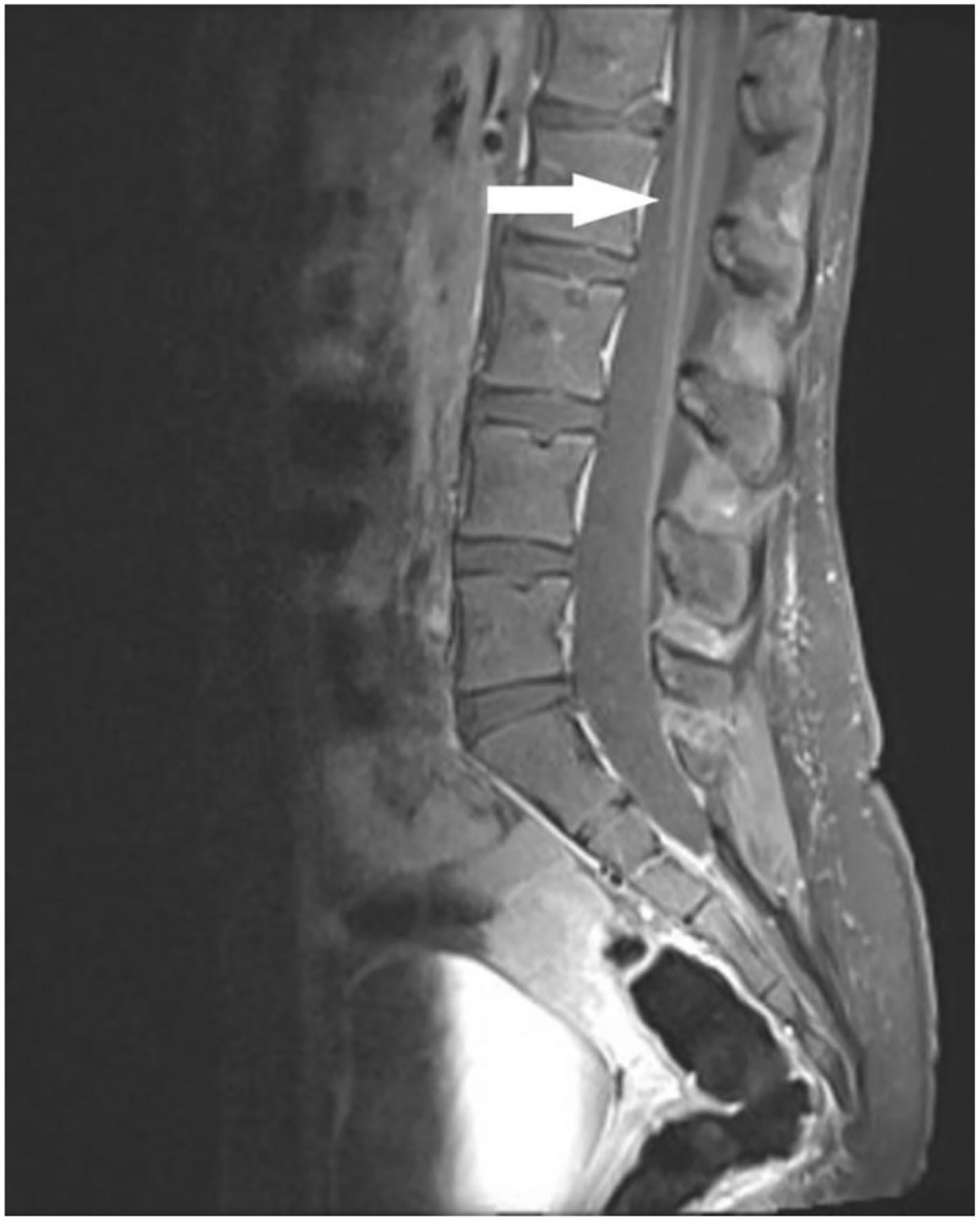
Sagittal post-contrast T1 weighted MRI image of lumbo-sacral spine demonstrating cauda equina root enhancement (arrow).

MRI of the spine showed normal cord width, signal morphology and intensity, and no syrinx or central spinal canal widening. There was mild enhancement of nerve roots in the cervical and thoracic regions, and mild thickening of the cauda equina nerve roots (Figure 2).

### EMG and Nerve Conduction Velocity

The nerve conduction study from Day 25 of admission showed significant reduction in compound muscle action potential (CMAP) amplitude in all four limbs with mild reduction in conduction velocity. Sensory conduction was normal. Overall, the findings were suggestive of acute motor axonal neuropathy typical of Guillain-Barré syndrome.

### Clinical Evolution and Therapy

**Figure 3:**
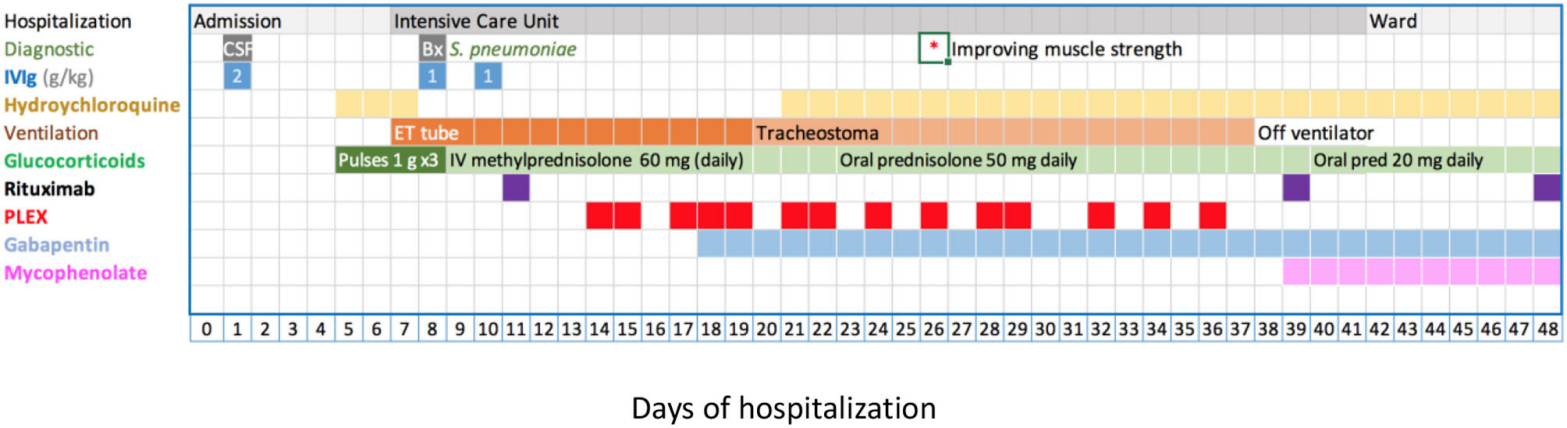
Disease evolution and management. Treatment details see text. Briefly, IVIG 2 g/kg as a single (day 1) or fractionated dose (days 8, 10), methylprednisolone 1 g/dose (20 mg/kg/dose), rituximab 375 mg/m^2^/dose. PLEX was against albumin and occasional units of fresh frozen plasma. CSF, cerebro-spinal fluid; ET, endotracheal tube; IVIG, intravenous immunoglobulin; PLEX, plasma exchange; *indicates the day, when an improvement of muscle strength was first documented.

Based on the Brighton criteria, the patient had the highest level of diagnostic certainty for acute inflammatory demyelinating polyneuropathy (GBS) (21). Treatment was initiated with 2 g IVIG per kg body in a single dose without clinical improvement, followed by three methylprednisolone pulses of 1 g each. However, her muscle weakness progressed, leading to hypoventilation. Spirometry showed a decline in vital capacity over the preceding 48 h from 25 to about 15 ml/kg, prompting elective endotracheal intubation and ventilation on day 7 of admission. Due to the lack of improvement following methylprednisolone pulses, IVIG, and subsequently rituximab, we started plasma exchange (PLEX) therapy on day 14 of admission (Figure 3).

Tracheostomy was performed on day 20 of admission in anticipation of prolonged invasive ventilation. Post tracheostomy analgosedation was weaned completely allowing neurological assessment of the wake patient and initiation of rehabilitative therapies. Of note, the patient experienced a brief episode of hyperactive delirium post tracheostomy.

The disease course was complicated by labile blood pressure, mostly hypertension, likely due to central autonomous dysregulation (9, 22). While intubated, she developed an uncomplicated, ventilator-associated *S. pneumoniae* pneumonia, diagnosed on day 9 of admission. The patient suffered from painful dysesthesia which improved with high-dose gabapentin and fentanyl boluses. Hair loss was noted during the early course of the disease. She received low molecular weight heparin (enoxaparin) during the period of paralysis.

PLEX treatment consisted of 14 sessions over 22 days, where 1-1.5 times the plasma volume was replaced with 5% albumin and 0.5 L fresh frozen plasma to maintain physiological coagulation factor concentrations. After about nine exchanges, the patient's muscle strength and respiratory efforts started to improve, and after the 12th session, we were able to gradually wean ventilator support.

The treatment was completed with two additional doses of rituximab on days 39 and 48 of admission. Peripheral CD19^+^ B cells was undetectable following the first rituximab infusion.

The patient was discharged home on Day 70 of admission with continued physiotherapy. At her first follow-up visit 4 days after discharge, she ambulated freely and performed all daily activities without assistance. Fourteen months after discharge, the patient maintains normal muscle strength. She was able to wean immunosuppression to hydroxychloroquine only. Peripheral CD19/20+ B cells started to reappear 9.5 months after the last rituximab dose, but remained low at 0.025/nL (N 0.200-0.600/nl). Serum levels of C3, C4 and anti-dsDNA antibodies, renal function and urinalysis were all normal.

## Discussion

SLE is a chronic autoimmune disease with multiple organ system involvement. Although lupus was not suspected initially, an antinuclear antibody panel and complement 3 and 4 concentrations were requested early after patient transfer to screen for an underlying etiology, particularly in view her family history with cases of SLE and (autoimmune) thyroid disease, respectively (Table 1). Recent infection or vaccination, including COVID-19, a newly recognized trigger of GBS or GBS spectrum (23–25), were ruled out (SARS-CoV-2 vaccines were not yet available at the time).

The clinical spectrum of SLE is variable and includes cutaneous, musculoskeletal, renal and neuropsychiatric manifestations. The American College of Rheumatology (ACR) research committee described twelve central (CNS) and seven peripheral nervous system (PNS) manifestations (including GBS) of neuropsychiatric SLE where other causes have been excluded (26). GBS can occur concomitantly with the initial presentation of SLE or years after the lupus diagnosis (15, 16, 18, 27). A large study from China (28) comprising 4,924 SLE patients, identified a subset of 73 patients (1.5%) with peripheral neuropathy, of whom a single patient presented with acute inflammatory demyelinating polyradiculoneuropathy (AIDP/GBS). A systematic review, summarizing data of 1,463 children and adolescents with SLE, identified 351 patients with NPSLE, two of whom presented with GBS (0.6% of all NPSLE patients) (5, 8). No clinical details are available from those cases. The pathophysiology of GBS in SLE patients remains speculative and is thought to involve lupus-induced auto-antibodies against myelin tissue (3, 29).

Treatment of GBS has been essentially unchanged over the past three decades (30). IVIg (2 g/kg divided over 5 days) or PLEX (5 sessions on alternate days (30–32) were shown to be equally effective treatment modalities for classical GBS, although some patients may respond slowly and/or achieve only partial resolution or may even worsen during conventional treatment (30). A combination of IVIg and PLEX has been advocated by some authors for recalcitrant cases of GBS (33). At variance with earlier EULAR treatment recommendations for SLE with neuropsychiatric manifestations (34), high-dose glucocorticoids alone have not shown benefit compared to placebo, nor in combination with IVIg or plasma exchange (30, 35, 36).

There are no published clinical trials or contemporary therapeutic guidelines for patients with SLE-GBS. Anecdotally, adult patient were treated with high-dose glucocorticoids, IV or oral cyclophosphamide, PLEX, IVIg, and mycophenolate mofetil, in various combinations (6, 7, 27, 29, 37, 38).

Current treatment recommendations for pediatric (non-SLE) GBS emphasize the use of IVIG over PLE (39, 40). However, this approach appears to be inadequate for patients with severe SLE-GBS as inferred from available experience in children and adults (5, 7, 16–18, 27, 29, 37, 38), including our patient.

The management of GBS patients in the critical (intensive) care unit (ICU), especially those requiring prolonged mechanical ventilation, can be challenging. The decision for tracheostomy is based on the expected duration of respiratory failure which may range from a few days to more than 6 months (41, 42). Early tracheostomy (43) helps avoid the drawbacks of long-term oral or nasal intubation and adequate sedation, and allows the patient to cooperate with functional assessment and physiotherapy.

We found only three previous descriptions of pediatric patients with SLE-GBS (see Table 2). Reddy et al. reported a 9-year-old girl from India who presented with progressive weakness over the preceding 4 days and a month after the onset of lupus symptoms (16). Treatment with IVIg, methylprednisolone pulses and IV cyclophosphamide failed to prevent progression to complete paralysis and intubation. She eventually improved after the second dose of rituximab. Javadi Parvaneh et al. described a 12-year-old Iranian boy with GBS, unresponsive to IVIg (17). He experienced a relapsing course with only partial improvement following high-dose glucocorticoid treatments. Interesting, anti-nuclear (ANA) and anti-dsDNA antibodies were noted at initial GBS presentation. He only achieved lasting GBS remission after commencing IV cyclophosphamide therapy in addition to IVMP pulses and hydroxychloroquine (17). The two cases differ from the scenario described in a singular earlier report of a 13-year-old Japanese teenager with “late onset SLE-GBS,” more than 6 years after the diagnosis of SLE and four months after a lupus flare, with ongoing cutaneous manifestations. He gradually recovered following PLEX, IVIg (0.4 g/kg), and high-dose glucocorticoid treatment (18) (Table 2).

**Table 2:** Literature review of pediatric SLE-GBS cases (attached separately).

**References**	**Demographics**	**Association between SLE and GBS manifestation**	**GBS severity**	**Kidney biopsy**	**Treatment**	**Treatment effect**	**Outcome**
**Early onset SLE-GBS**							
Reddy et al. (16)	9 y Female	Acute motor axonal polyradiculoneuropathy 4 weeks after onset of malar rash and hair loss. SLE serology pos at GBS presentation, incl. anti-SSA/SSB AGA (GM2, GM3) pos FH not reported	Respiratory failure, intubation and ventilation DA10, ICU stay 27 d	ND	DA 2-6 IVIg 2g/kg div 5d IVMP 30 mg/kg x5, then thrice weekly IVCY (×1) DA6 RTX (×2) DA20,34	Progressive resp failure, intubation DA10 Vent D10-42	Full remission at 12 mo SLE controlled with HCQ only
Javadi Parvaneh et al. (17)	12 y Male	GBS/AIDP → CIDP Lupus serology pos at presentation SLE diagnosis 4 mo after GBS onset AGA (Anti-GM) pos FH of SLE (sister)	Prolonged, relapsing course of muscle weakness over several mo without respiratory insufficiency	ND	IVIg IVMP ×5 OP ×3 mo SLE treatment IVMP, HCQ, IVCY	Ineffective Partial response and relapse GBS Improvement w/ IVCY	Complete neurological recovery after several mo
Beshir et al. (this report)	14 y female	No preceding illness SLE serology pos at GBS manifestation incl. anti-SSA/B Subtle, non-specific clinical lupus AGA (GM1) neg FH of SLE (aunt)	Progressive ascending, weakness over 4 weeks, dysphagia, diplopia prior to admission, mild temp at presentation ICU stay 34 d	LN WHO class II (during acute GBS)	IVIg 2g/kg DA3,11 IVMP 1g ×3 DA5,7,8, then 60 mg/d and taper RTX 3 ×375 mg/m^2^ DA11,39,48 PLEX (14) DA14-36 MMF D39 ×2 mo HCQ D21 (continued)	IVIg, GC ineffective Gradual response after 10 PLEX, Vent D7-37	Complete GBS recovery SLE remission B cell count still largely suppressed 15 mo after last dose
**Late-onset SLE-GBS**							
Miyagawa et al. (18)	13 y female	Onset of SLE and Sjögren's age 7y (anti-SSA/B pos). SLE flare at 13y, controlled w/ >OP Acute GBS onset 4 mo after lupus flare with active cutaneous lupus AGA (myelin glycolipid) neg FH not reported	Respiratory failure, intubation and ventilation ICU stay 27 d	Unremarkable (during lupus remission, prior to GBS)	PLEX (13), then IVIg 0.4 g/kg ×3 and high-dose glucocorticoids (IVMP 1g ×3, then OP)	Gradual resolution	Complete remission 5 mo after GBS onset SLE controlled with low-dose OP

*Medication doses, PLEX sessions or kidney biopsy results are given as (and if) reported in the referenced publications. Ab, antibody; AGA, anti-ganglioside antibodies; AIDP, Acute inflammatory demyelinating polyradiculoneuropathy; CIDP; chronic inflammatory demyelinating polyradiculoneuropathy; d, day(s); DA, day(s) of admission; FH, family history; GBS, Guillain-Barré syndrome; HCQ, hydroxychloroquine; ICU, intensive care unit; IVCY, intravenous cyclophosphamide; IVIg, intravenous immunoglobulin; IVMP, intravenous methylprednisolone (pulse); LN, lupus nephritis; MMF, mycophenolate mofetil; mo, month(s); ND, not done; OP, oral prednisolone; PLEX, plasma exchange therapy; RTX, rituximab; Vent, intubated and ventilated; y ,year(s)*.

The strengths of the current paper are the detailed diagnostic workup and timely interdisciplinary care, and a synoptic review of all accessible pediatric cases. Limitations are those inherent in (anecdotal) case presentations.

In conclusion, our report highlights the importance of prompt recognition of SLE as a trigger of GBS, which changes conventional GBS management. Due to the rarity of SLE-GBS within the GBS spectrum, specific treatment recommendations are lacking. It is plausible to target the underlying autoimmune disorder (SLE) and the removal of presumed pathological antibodies and/or suppression of harmful proinflammatory effects. B cell depleting agents and plasma exchange are potentially effective strategies. While glucocorticoids have no proven role in the treatment of GBS, their administration in SLE-GBS as part of the control of lupus needs be redefined. The dissemination of further experiences and studies is warranted to establish therapeutic efficacies and outcomes for children presenting with GBS in the context of SLE.

## Data Availability Statement

The datasets generated for this study are available on request to the corresponding author.

## Author Contributions

EBes participated in the care of the patient, collected data, reviewed the literature, and wrote the manuscript. EBel supervised the medical treatment and revised the manuscript. NC and AA supervised and interpreted the neurological aspects of the diagnosis and treatment, and contributed to the manuscript. AS, MP, and UT supervised the patient's treatment in the critical care unit and contributed to the manuscript. LH read and interpreted the kidney biopsy and provided the photographs. ES provided expert rheumatological advice. MB contributed to the patient's medical and renal care, reviewed and interpreted the clinical and laboratory data, and wrote and edited the manuscript. All authors critically reviewed the manuscript and agreed to the final version.

## Conflict of Interest

The authors declare that the research was conducted in the absence of any commercial or financial relationships that could be construed as a potential conflict of interest.

## Publisher's Note

All claims expressed in this article are solely those of the authors and do not necessarily represent those of their affiliated organizations, or those of the publisher, the editors and the reviewers. Any product that may be evaluated in this article, or claim that may be made by its manufacturer, is not guaranteed or endorsed by the publisher.

## Abbreviations

CNS: central nervous system
CSF: cerebrospinal fluid
GBS: Guillain-Barré syndrome
IVIg: intravenous immunoglobulin
NPSLE: neuropsychiatric systemic lupus erythematosus
PLEX: plasma exchange
PNS: peripheral nervous system
SLE: systemic lupus erythematosus
SLE-GBS: GBS due to SLE.
